# Allocation trade-off under climate warming in experimental amphibian populations

**DOI:** 10.7717/peerj.1326

**Published:** 2015-10-20

**Authors:** Xu Gao, Changnan Jin, Arley Camargo, Yiming Li

**Affiliations:** 1 Key Laboratory of Animal Ecology and Conservation Biology, Institute of Zoology, Chinese Academy of Sciences, Beijing, China; 2 University of Chinese Academy of Sciences, Beijing, China; 3 Chinese National Geography Magazine, Beijing, China; 4 Centro Universitario de Rivera, Universidad de la República, Rivera, Uruguay

**Keywords:** Trade-off, Allocation strategy, Reproduction, Climate change, Food availability, Experimental warming, Feeding rate, Phenology, Mesocosm, Climate warming

## Abstract

Climate change could either directly or indirectly cause population declines via altered temperature, rainfall regimes, food availability or phenological responses. However few studies have focused on allocation trade-offs between growth and reproduction under marginal resources, such as food scarce that may be caused by climate warming. Such critical changes may have an unpredicted impact on amphibian life-history parameters and even population dynamics. Here, we report an allocation strategy of adult anuran individuals involving a reproductive stage under experimental warming. Using outdoor mesocosm experiments we simulated a warming scenario likely to occur at the end of this century. We examined the effects of temperature (ambient vs. pre-/post-hibernation warming) and food availability (normal vs. low) on reproduction and growth parameters of pond frogs (*Pelophylax nigromaculatus*). We found that temperature was the major factor influencing reproductive time of female pond frogs, which showed a significant advancing under post-hibernation warming treatment. While feeding rate was the major factor influencing reproductive status of females, clutch size, and variation of body size for females, showed significant positive correlations between feeding rate and reproductive status, clutch size, or variation of body size. Our results suggested that reproduction and body size of amphibians might be modulated by climate warming or food availability variation. We believe this study provides some new evidence on allocation strategies suggesting that amphibians could adjust their reproductive output to cope with climate warming.

## Introduction

Climate change is predicted to play a crucial role in population declines and loss of diversity (Thomas et al., 2004). Under current climate warming, the responses of reproductive traits in many animals have attracted more global attention in recent research. Several examples included the mis-timing in reproduction under climate change in birds (Visser, Both & Lambrechts, 2004), the reproductive performance of the common lizard (*Lacerta vivipara*) affected by rainfall regime (Marquis, Massot & Le Galliard, 2008), and an advanced reproductive time and increased clutch size under climate warming in the Chinese alligators (*Alligator sinensis*) (Zhang et al., 2009). As sensitive taxa, amphibians have been declining worldwide and some of them are facing a real threat of extinction (Pechmann et al., 1991; Houlahan et al., 2000). With the continuing trend of global warming, there may be increasing unpredictable impacts on amphibian fitness (Gouveia et al., 2013; Visser, 2013).

Climate change could either directly change amphibian survival rate and reproductive success via altered temperature and rainfall regimes, or indirectly, due to limited or increased food availability, or phenological responses (Carey & Alexander, 2003; Gouveia et al., 2013; Høye et al., 2014). Several studies have performed control experiments to obtain further information for assessing the impact of future climate warming on both amphibian larvae and adults (Garner, Rowcliffe & Fisher, 2011; O’Regan, Palen & Anderson, 2014). However, there were few experiments examining the allocation trade-off between growth and reproduction of marginal resources, which could affect life-history parameters and even population dynamics (Li, Cohen & Rohr, 2013). This is especially true for meso-scale experiments at a semi-natural state involving the allocation strategy for adult individuals under climate warming scenarios (Roff, 1992; Stewart et al., 2013). As ectothermic animals, amphibians have a typical positive relationship between metabolic rate and temperature (Gillooly et al., 2001; Dillon, Wang & Huey, 2010). For instance, the common toad (*Bufo bufo*) does not hibernate when exposed to a warmer temperature during the hibernation period (Jørgensen, 1986; Reading, 2007). Toads that did not hibernate showed lower growth rates, smaller body size at maturity, and higher mortality than ones that experienced hibernation, when food resources are provided *ad libitum* (Jørgensen, 1986; Reading, 2007). In addition, in a suitable temperature range, metabolic and developmental rates of amphibian larva increased with temperature (Álvarez & Nicieza, 2002; Narayan & Hero, 2014). A shorter larval period may lead to smaller size at metamorphosis under a fixed amount of resources due to a higher metabolic demand (Enriquez-Urzelai et al., 2013). However, an extreme thermal environment could also cause harmful effects for adult *Rhinella marina* (Narayan & Hero, 2014). Based on this evidence, we would expect that climate warming would cause an overall fitness reduction in amphibian populations even when food resources remain constant.

Climate change could also affect the food resources of many animals either in phenophase or in quantity (Huston & Wolverton, 2011; Visser, 2013). These variations in food resources may trigger unpredictable effects on phenophase, body condition, survival and reproduction (Reading, 2007; Gouveia et al., 2013; Visser, 2013; Fox et al., 2014). For instance, reproduction in birds can mismatch the phenology of their insect prey, plants can decrease their reproductive capacity as the result of increased insect abundance, and fish may miss the plankton’s peak abundance (Kristiansen et al., 2011; Liu et al., 2011; Visser, 2013). On the other hand, an abundant food resource would lead to bigger clutch sizes and nestlings in birds (Arcese & Smith, 1988), and more cubs in mammals (Angerbjörn et al., 1991). In addition, the eastern fence lizard (*Sceloporus undulatus*) has an ability to flexibly allocate resources between reproduction and growth to deal with the fluctuation of food resources (Warne et al., 2012). Such variations in food resources may also affect the fitness of amphibians, which is shown in a controlled experiment with the common frogs (*Rana temporaria*) revealing that both the females’ growth and reproduction respond positively to high food rations (Lardner & Loman, 2003). Based on such evidence, we predict that there should be a negative effect on amphibian fitness when food availability is reduced as a consequence of climate warming.

As animals with indefinite (indeterminate) growth, amphibians have to allocate their energy to survive, grow and reproduce (Kozlowski, 1996; Kozlowski & Teriokhin, 1999). Therefore, given a certain amount of energy, there is a trade-off between reproduction at the present and investment in growth for future reproduction, which should lead to an optimal allocation pattern for higher fitness (Roff, 1992; Kozlowski, 1996; Kozlowski & Teriokhin, 1999). In addition, the large-sized individuals have better fitness in reproduction than small ones in most amphibians (Duellman & Trueb, 1986; Wells, 2010). If the resources were invested in growing a larger body size, the individual would produce more offspring in the future. In the extreme case, the animal could distribute all surplus energy to growth and survival in order to avoid a period of harsh conditions for reproduction, or they would invest in reproduction to avoid disadvantages that might appear in the following seasons (Schwarzkopf, 1993; Roff, 2002). Some animals, such as *S. undulatus* have evolved an allocation strategy between growth and reproduction (Warne et al., 2012). However, how the allocation strategy between growth and reproduction would change under the current climate warming is unclear. Therefore, a controlled experiment on allocation strategy is an appropriate method that may be able to establish a mechanism generating the trade-off (Roff, 1992).

The pond frogs (*Pelophylax nigromaculatus*) is a common large-sized anuran species (mean snout-vent length ∼62 mm for males and ∼74 mm for females) widely distributed in Eastern Asia (Fei, 1999). The species is documented as reproducing once every year without parental care of eggs or tadpoles (Fei, 1999; Wang et al., 2009). It is an ideal animal to examine the changes of reproductive strategies and trade-offs for our study, since using it excludes other potential confounding effects, such as multiple clutches in a single breeding season and parental care of offspring.

In order to predict the potential effects of climate warming on life-history trade-offs of amphibians, it is necessary to implement appropriate experimental studies by controlling the interaction between temperature and food resources. In comparison with these fieldwork-based studies, outdoor mesocosm experiments could distinguish between the effects due to climate warming itself, and the variation in food availability caused by climate warming (Stewart et al., 2013). Hence, we performed a cross-year study using non-destructive measurement techniques to examine the effect of temperature scenarios (ambient vs. pre-/post-hibernation warming) and food levels (normal vs. low) on reproductive parameters and allocation strategies for reproduction. Our mesocosm setting imitated the microhabitats of this species and performed experimental warming under daily temperature fluctuations with an average of around 2.3 °C warmer than ambient temperature. Our study addressed the following questions: (1) whether there existed changes in reproductive parameters under these simulated scenarios of climate warming, (2) whether there existed a trade-off in allocating limited food for growth or reproduction on females, and (3) whether the allocation strategies were affected by temperature or feeding rate.

## Materials and Methods

### Ethics statement

The experiments were conducted under the approval of the Animal Care and Ethics Committee and carried out in accordance with the guidelines for the Use of Animals in Research issued by Institute of Zoology, Chinese Academy of Sciences (Project No. 2011/43).

### Study background

The experiments were conducted in Yanchang Village, Taohua town (29.85°N, 122.26°E), northeast Zhejiang Province, China. The climate is typical of subtropical monsoonal regime with clear seasons, a mean annual temperature of 15.6–16.6 °C, and a mean annual precipitation of 936.3–1,330.2 mm (Zhoushan City Government, 1992). This frog is a dominant and common anuran species in this area, which generally reproduces from April to June in this region and usually spawns near submerged vegetation in shallow waters (Wang et al., 2007; Wang et al., 2009).

### Experimental methods

**Figure 1:**
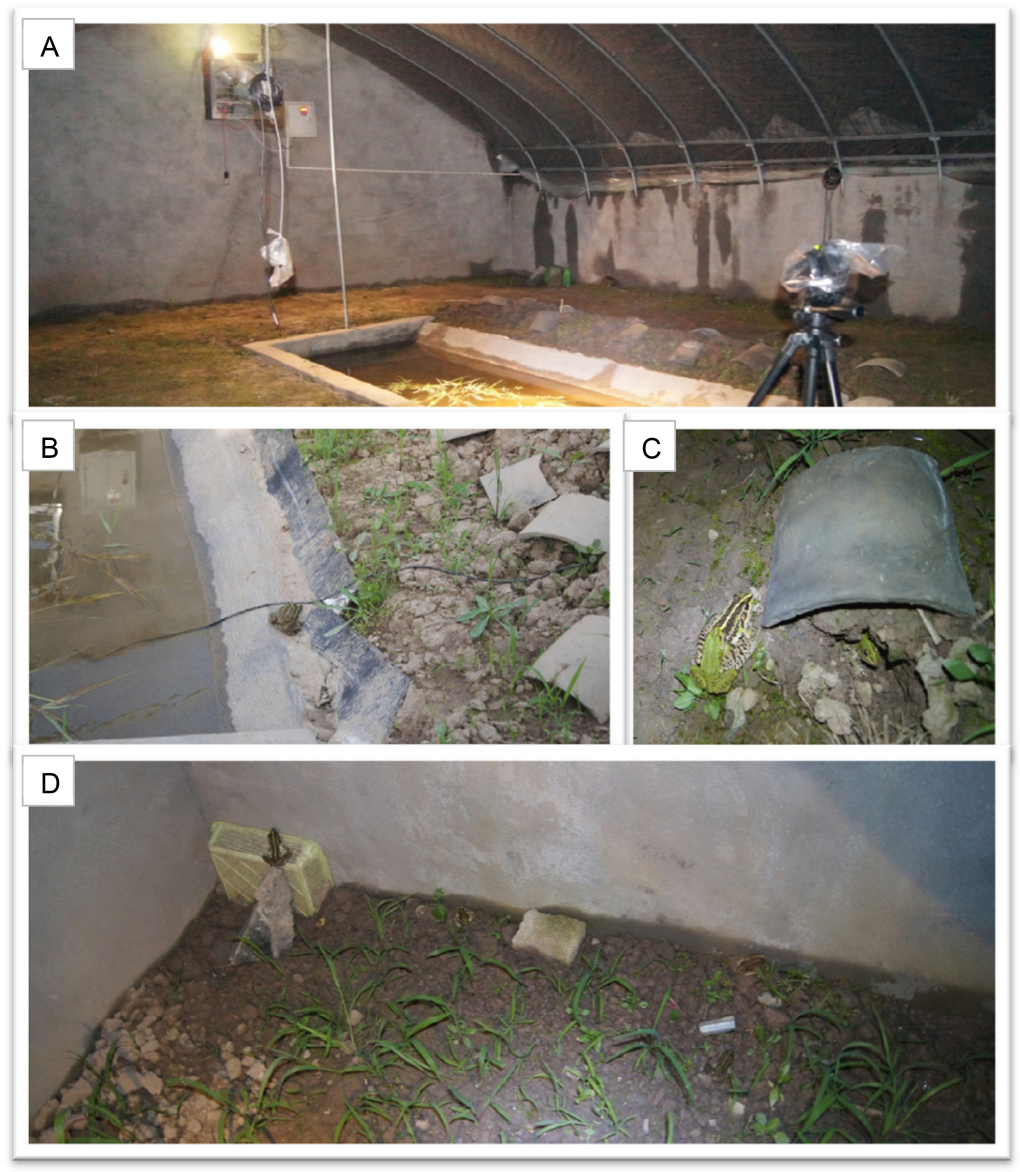
The experimental photos. (A) The interior of an experimental warming mesocosm unit; (B) a pair of mating pond frogs and the typical hiding sites for them; (C) the close-up of a pair of frogs and a typical hiding site; (D) the environment of a mesocosm corner in the experiment. Photo credits: Xu Gao.

We performed the study during 14th Oct 2011 to 14th Jul 2012 using 18 mesocosm units. Each of them was approximately 45.5 m^2^ (7 × 6.5 m), surrounded by walls of bricks (height 1.2 m). Each unit was covered by an arc-shaped steel frame covered with plastic films (during warming period) or insect screens (during other experimental period). We simulated the typical habitat of the species in each unit: the soil was substituted with deep-lay soil and floored with grass, a 3.6 m^2^ pond (3 × 1.2 m and depth 0.5 m) was dug at the middle of each unit. We provided refuges for frogs such as gaps around bricks, stones or tiles, and the frogs could also make holes on the soft ground by themselves (Fig. 1).

**Figure 2:**
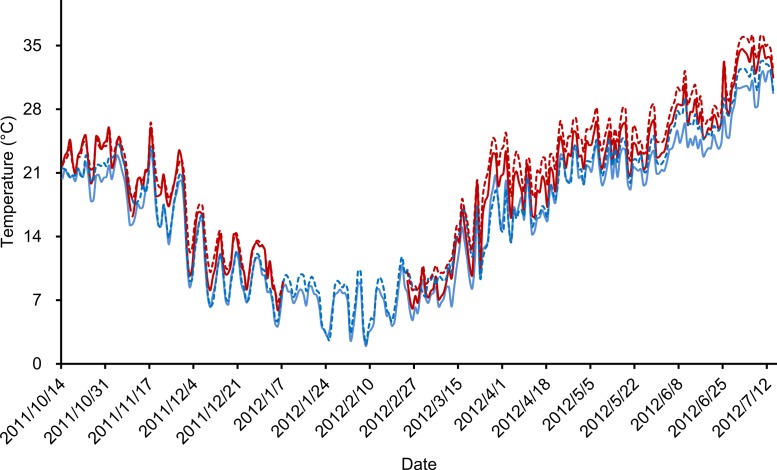
Daily average temperatures (°C) recorded during experimental period. Solid lines indicated the air temperature and dotted lines indicated the water temperature, blue color indicated ambient temperature treatments and red color indicated experimental warming treatments.

Our outdoor mesocosm experiments were designed with three temperature scenarios (ambient temperature, pre- and post-hibernation warming) and two food levels (normal and low). In total, six treatments were deployed: (i) control group, with ambient temperature and normal food level (CG), (ii) low food treatment, with ambient temperature and low food level (LF), (iii) pre-hibernation warming treatment, with normal food level (PreW), (iv) pre-hibernation warming and low food treatment (PreW-LF), (v) post-hibernation warming treatment, with normal food level (PostW), and (vi) post-hibernation warming and low food treatment (PostW-LF). Each treatment was replicated in 3 mesocosms, since our experiment designed as semi-natural study, the confined space of each mesocosm unit could not hold too many frogs. The pre-hibernation warming treatments (PreW and PreW-LF) were heated for 85 days (from 14th Oct 2011 to 7th Jan 2012, lasting 20 days; after which, all the frogs naturally went into hibernation) (Gao et al., 2015); analogously, the post-hibernation warming treatments (PostW and PostW-LF) were heated for 141 days (from 24th Feb 2012 to 14th Jul 2012, starting with 3 days of air temperature >8 °C, and lasting 60 days after the last natural spawning behavior). Both pre- and post-hibernation warming treatments used the greenhouse method (Gao et al., 2015). The air temperatures of pre- and post-hibernation warming periods were automatically regulated by real-time monitoring equipment that simulated a daily fluctuating temperature regime (Gao et al., 2015). We recorded air and water temperature of each mesocosm every hour using data-loggers (Jingchuang RC-500+) during the experimental periods. The air temperature was defined as the temperature 1 m above the ground surface, and the water temperature was defined as the temperature 10 cm below the water surface. The air and water temperature were highly correlated in these treatments (Pearson correlation test, ambient temperature: *r* = 0.991, *P* < 0.001; experimental warming: *r* = 0.984, *P* < 0.001; Fig. 2). Finally, we warmed the mesocosm 2.2–2.4 °C above the ambient temperature in pre-/post-hibernation warming period. The increases of temperature are consistent with the prediction range (1.8–4.0 °C) of global average surface temperature by the end of the century (IPCC, 2007).

In mid-October 2011, 270 frogs were captured from the wild in the town of Taohua and assigned to each mesocosm randomly (approximately 6 males and 9 females per mesocosm). Here, we acknowledged that the frogs were collected from one site, despite the fact that the clutch size of this species changes among different sites in the wild (Fei, 1999; Wang et al., 2007; Wang et al., 2009). Geographic and temporal variations in reproductive parameters are common phenomena in amphibians (Sparks et al., 2007; Camargo, Sarroca & Maneyro, 2008; Green & Middleton, 2013). However, our experiment is dealing with the change of reproductive parameters and allocation trade-off patterns, meaning that there should be qualitatively similar composition of individuals among treatments. Only then can we examine the changing trends of these frogs under experimental conditions (temperature and food) rather than those variations in geographic and temporal dimensions. In addition, we ignored the potential for diseases among experimental frogs, such as chytrids, since there is lack of evidence for risk of diseases in this area (Bai et al., 2012; Zhu et al., 2014a; Zhu et al., 2014b).

During the experiment, the frogs were artificially fed with standard commercial crickets one by one (*Gryllus bimaculatus*, approximately 15 mm long). The crickets were dusted with calcium powder and provided every night for the normal food level treatments (CG, PreW and PostW) and every 3 days for the low food level treatments (LF, PreW-LF and PostW-LF; equaling to 1/3 of the normal food level treatments; Leips & Travis, 1994). All frogs were divided into four weight classes (≤20 g, 20–40 g, 40–60 g and >60 g) at the beginning and supplied with corresponding cricket numbers 1:2:3:4 (Lardner & Loman, 2003). Each frog was assigned 1-minute feeding time since they preyed on the crickets immediately, after which we cleared uneaten crickets and recorded the information (Gao et al., 2015).

At the beginning of the experiment, each frog was measured. Then, they were marked with built-in PIT-tags (2 mm × 8 mm, HongTeng HT950) on the right thighs and each individual could be identified by a hand-held scanner (Kingdoes KD-Pi60) with unique identification code of its tag. During the experiment, we did not artificially induce the frogs to hibernation or reproduction, we only provided suitable environment for frogs and they were spontaneously entered into/emerged from hibernation and bred as they were in the wild. During post-hibernation warming period, the reproductive behaviors of frogs were observed every six hours (6:00, 12:00, 18:00 and 24:00), hence we could immediately record the breeding time, measure clutch size (egg number) and egg size (to reduce the human disturbance, the diameters of 100 eggs were measured, which were randomly sampled per clutch). After the eggs hatched, we released the tadpoles at the place where their parents were captured. At the end of our experiment, we measured the snout-vent length (SVL) and weight of the frogs and estimated their age by skeletochronology (Li et al., 2011), then we released all the frogs too.

### Statistical analyses

After the experiment, we only use the data from females for this research. Given fact that male frogs may mate several times or indulge in post-mating clutch piracy (Vieites et al., 2004), it was difficult to determine which individuals actually reproduced in our experiment. We used Kolmogorov–Smirnov tests for detecting normality and Levene’s tests to test for heterogeneity of variances. We logarithmically transformed the variances to minimize the heterogeneity if necessary (Zar, 2009). We defined reproductive time as the day number when the event occurred between the 1st of January (as day 1) and the 31th of December (as day 365; Visser & Both, 2005). We used a composite index of body size reflecting both SVL and weight based on the principal component analysis (PCA) scores of the SVL and weight data (see details in Lardner & Loman, 2003). Then, we used the variation of body size to reflect both changes in SVL and weight during the experiment, which was calculated as the difference between final and initial body size.

Because the parameter food level could not reflect the food availability of each individual, we used an index of feeding rate (crickets consumed per individual frog). The feeding rate was defined as the residual in a standardized major axis (SMA) regression of crickets consumed on original weight, using the *smatr* package for R software (Lardner & Loman, 2003; Warton et al., 2006; Warton & Ormerod, 2007). Because the metabolic rate may vary with temperature (Gillooly et al., 2001), we computed this index in each temperature scenario (hereafter, we used the feeding rate to represent the food level).

We examined the differences in reproductive status of individuals (reproductive or non-reproductive) in these treatments using binary logistic regression model with temperature, feeding rate, temperature × feeding rate, and initial body size as covariates. Further, we examined differences in reproductive time, clutch size, egg size, and the variation in body size among these treatments using analysis of covariance (ANCOVA) with temperature as a fixed factor, and feeding rate, temperature × feeding rate and initial body size (this variable might have effects on these reproductive parameters) as covariates. In addition, we used multiple linear regression models to examine the relationships among reproductive time, clutch size, egg size, and the variation in body size with temperature, feeding rate, temperature × feeding rate, and initial body size as predictors.

Clutch size investment was defined as the residual of clutch size on original body size by a SMA regression. Similarly, the egg size investment was calculated as the residual of egg size on original body size by a SMA regression. We examined differences in clutch size investment and egg size investment among these treatments using ANCOVA with temperature as a fixed factor, and feeding rate and temperature × feeding rate as covariates. After that, we calculated the residual of clutch size investment on egg size investment by a SMA regression and examined the residual for testing the clutch size/egg size investment trade-off by ANCOVA with temperature as fixed a factor, feeding rate, feeding rate × temperature as covariates. Then, we used the residual of a regression between clutch volume (*π*/6 × *Egg size*^3^ × *Clutch size*) and initial body size as a measure of reproduction investment index for these females that have laid eggs successfully. The reproduction investment index was calculated as the residual of clutch volume on original body size by a SMA regression. In addition, we used the residual of a SMA regression as the growth investment index, which was computed as final body size on original body size. We examined differences in reproduction investment and growth investment among these treatments using ANCOVA with temperature as a fixed factor, and feeding rate and temperature × feeding rate as covariates. Finally, we calculated the residual of reproduction investment on growth investment by a SMA regression and examined the residual for testing the reproduction/growth investment trade-off by ANCOVA with temperature as fixed factor, feeding rate, feeding rate × temperature as a covariates. Results were considered significant if *P* ≤ 0.05. All analyses were conducted using R v3.1.2 (R Development Core Team, 2012).

## Results

**Figure 3:**
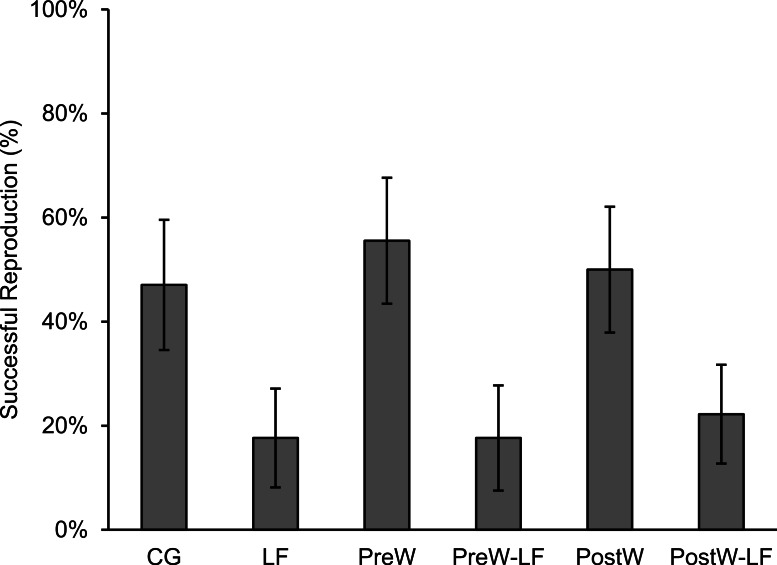
The successful reproduction percentage of female *P. nigromaculatus* (Mean ± SE) among experimental treatments. Control group (CG), low food treatment (LF), pre-hibernation warming with normal (PreW) or low food level treatment (PreW-LF), post-hibernation warming with normal (PostW) or low food level treatment (PostW-LF).

In total, 105 females survived and were included in our analyses, of which 37 individuals bred successfully. As our statistical analyses were related to initial body size, it was necessary to distribute initial body size equally among treatments. Our results showed that the following three body size classes were not significantly different among treatments: small (≤ − 0.5, *n* = 40; one-way ANOVA, *F* = 0.923, *df* = 5, *P* = 0.478), middle (−0.5–0.5, *n* = 39; *F* = 1.036, *df* = 5, *P* = 0.413) and large (≥0.5, *n* = 26; *F* = 1.548, *df* = 5, *P* = 0.220). The reproductive parameters of female *P. nigromaculatus* were highly variable among treatments. The individuals that bred successfully in the experimental treatments were from 17.7% (LF and PreW-LF, respectively) to 55.6% (PreW; Fig. 3). The reproductive time varied from day 90 (PostW) to day 127 (CG; Table 1). Clutch size ranged between 607 (PostW) and 4,553 (CG), while the egg size varied between 1.47 mm (PreW) and 1.64 mm (PostW; Table 1).

**Table 1:** A summary of female *P. nigromaculatus* and reproductive parameters among experimental treatments. Control group (CG), low food treatment (LF), pre-hibernation warming with normal (PreW) or low food level treatment (PreW-LF), post-hibernation warming with normal (PostW) or low food level treatment (PostW-LF).

Treatment	*n*	Age (year)	Reproduced individual	Reproductive time (day)	Clutch size	Egg size (mm)
CG	17	2.4 ± 0.4	8	118.6 ± 2.3	1,804 ± 493	1.58 ± 0.02
		(1–6)		(106–127)	(723–4,553)	(1.51–1.63)
LF	17	2.4 ± 0.3	3	118.3 ± 4.1	1,195 ± 196	1.56 ± 0.02
		(1–5)		(112–126)	(851–1,528)	(1.53–1.59)
PreW	18	2.9 ± 0.4	10	114.6 ± 2.3	2,133 ± 354	1.55 ± 0.01
		(1–6)		(105–125)	(811–4,262)	(1.47–1.62)
PreW-LF	17	2.5 ± 0.4	3	116.3 ± 2.9	1,166 ± 239	1.55 ± 0.02
		(1–6)		(111–121)	(783–1,606)	(1.53–1.60)
PostW	18	2.6 ± 0.4	9	106.9 ± 3.3	1,604 ± 411	1.58 ± 0.02
		(1–6)		(90–122)	(607–4,104)	(1.49–1.64)
PostW-LF	18	2.6 ± 0.4	4	102.0 ± 4.8	1,298 ± 188	1.56 ± 0.01
		(1–6)		(92–115)	(782–1,590)	(1.54–1.60)

The binary logistic regression showed that there were significant and positive relationships between the reproductive status of female *P. nigromaculatus* and feeding rate (*β* = 0.014, *P* = 0.031), and initial body size (*β* = 5.256, *P* < 0.001). However, there was no significant relationship between reproductive status and either temperature (*P* = 0.153) or temperature × feeding rate interaction (*P* = 0.759) among the treatments.

**Figure 4:**
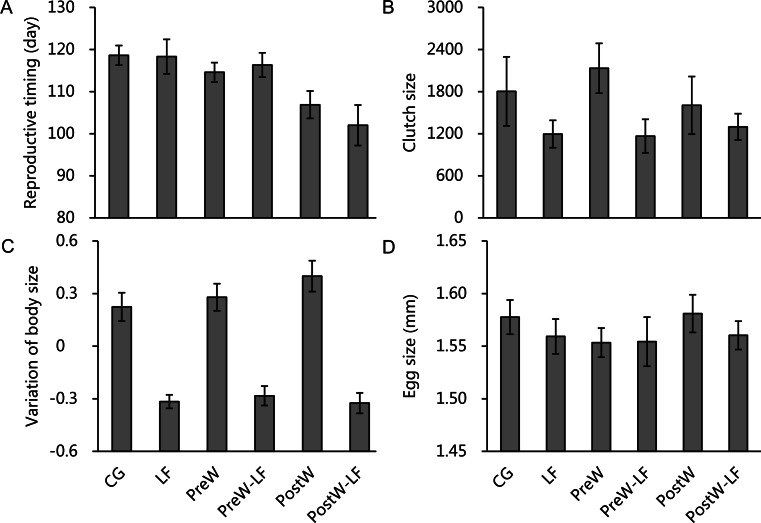
The reproductive timing (A), clutch size (B), variation of body size (C) and egg size (D) of female *P. nigromaculatus* (Mean ± SE) among experimental treatments. Control group (CG), low food treatment (LF), pre-hibernation warming with normal (PreW) or low food level treatment (PreW-LF), post-hibernation warming with normal (PostW) or low food level treatment (PostW-LF).

Because the random factors (replicates) were not significant (Table S1), we removed it from the ANCOVA models in order to increase statistical power. The ANCOVAs showed that reproductive time, clutch size and the variation in body size for female *P. nigromaculatus* differed among experimental treatments (Figs. 4A–4C), but egg size did not (Fig. 4D). After controlling for initial body size, temperature had a significant effect on reproductive time, and feeding rate significantly affected clutch size and the variation of body size, but temperature, feeding rate and interaction did not show any significant effect on egg size (Table 2). Pairwise comparisons indicated that the post-hibernation warming treatments had an earlier reproductive time than the other two temperature scenarios (Bonferroni, vs. ambient temperature treatments: *P* < 0.001; vs. pre-hibernation warming treatments: *P* = 0.002; Fig. 4A), while there was no significant difference between ambient temperature and pre-hibernation warming treatments (Bonferroni, both of them: *P* = 1.000; Fig. 4A). There were significant, positive relationships between feeding rate and clutch size (*β* = 0.001, *t* = 2.298, *P* = 0.029; Fig. 4B), and the variation of body size (*β* = 0.003, *t* = 4.903, *P* < 0.001; Fig. 4C). Moreover, the models also indicated initial body size have significantly negative relationships with reproductive time (*β* = − 3.899, *t* = − 2.622, *P* = 0.014) and egg size (*β* = − 0.043, *t* = − 12.717, *P* < 0.001), while there was a significantly positive relationship with clutch size (*β* = 0.290, *t* = 22.288, *P* < 0.001), but no relationship with the variation of body size (*P* = 0.431). Further details of the variations of weight and SVL in supporting information are provided in Table S2.

**Table 2:** Summary of ANCOVAs for the reproductive parameters and growth of female *P. nigromaculatus* in mesocosm experiments. With temperature as a fixed factor, and using feeding rate, temperature × feeding rate and initial body size as covariates.

Source of variation	Reproductive time (day)		Clutch size (log_10_-transformed)		Egg size (mm)		Variation of body size	
	*df*	*F*	*df*	*F*	*df*	*F*	*df*	*F*
Temperature	2	11.829^***^	2	0.209	2	0.863	2	0.574
Feed rate	1	0.034	1	38.425^***^	1	1.589	1	68.103^***^
Temperature × feeding rate	2	0.375	2	0.849	2	0.837	2	0.571
Initial body size	1	6.875^*^	1	496.769^***^	1	161.735^***^	1	0.626
Error	30		30		30		98	

**Notes.**

* *P*-value < 0.05 (2-tailed).

*** *P*-value < 0.001 (2-tailed).

**Figure 5:**
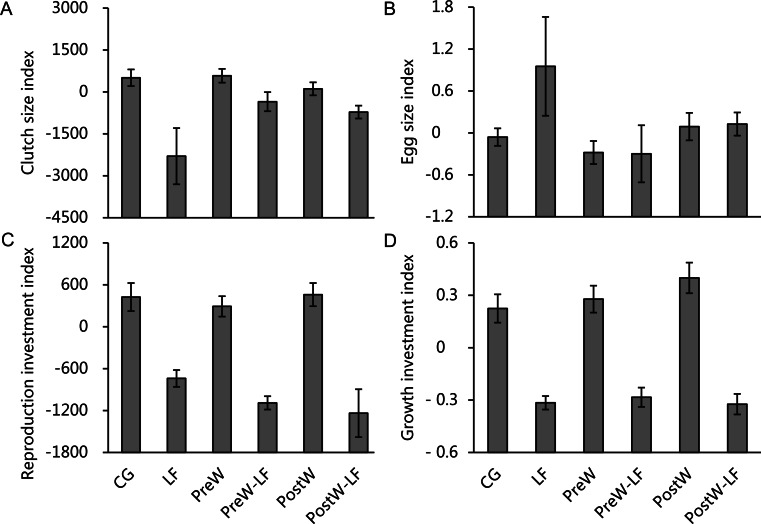
The investment index of clutch size (A), egg size (B), reproduction (C) and growth (D) of female *P. nigromaculatus* (Mean ± SE) among experimental treatments. Control group (CG), low food treatment (LF), pre-hibernation warming with normal (PreW) or low food level treatment (PreW-LF), post-hibernation warming with normal (PostW) or low food level treatment (PostW-LF).

The ANCOVAs showed that only feeding rate had significant effects on clutch size investment, reproduction investment and growth investment for female *P. nigromaculatus* among experimental treatments, but there was no significant effect on egg size investment (Table 3). The model showed that there was a significantly positive relationship between feeding rate and clutch size index (*β* = 8.914, *t* = 3.064, *P* = 0.004; Fig. 5A), reproduction investment index (*β* = 4.219, *t* = 3.427, *P* = 0.002; Fig. 5C) and growth investment index (*β* = 0.003, *t* = 4.855, *P* < 0.001; Fig. 5D), but there was no significant relationship between feeding rate and egg size index (*t* = − 1.971, *P* = 0.058; Fig. 5B)

In allocation trade-off analyses, the ANCOVA showed that feeding rate had significant effects on clutch size/egg size investment trade-off, but temperature and feeding rate × temperature interaction did not (Table 4). There was a significantly positive relationship between feeding rate and the clutch size/egg size investment trade-off (*β* = 2.947, *t* = 2.089, *P* = 0.045). However, the ANCOVA showed that none of these factors has effect on reproduction/growth investment trade-off (Table 4). More details separated by different age classes are provided in Figs. S1–S3.

**Table 3:** Summary of ANCOVAs for the clutch size investment, egg size investment, reproduction investment and growth investment of female *P. nigromaculatus* in mesocosm experiments. With temperature as a fixed factor, using feeding rate and temperature × feeding rate as covariates.

Source of variation	Clutch size investment		Egg size investment		Reproduction investment		Growth investment	
	*df*	*F*	*df*	*F*	*df*	*F*	*df*	*F*
Temperature	2	1.021	2	2.236	2	0.748	2	0.601
Feed rate	1	7.815^**^	1	0.343	1	74.776^***^	1	75.295^***^
Temperature × feeding rate	2	2.579	2	2.477	2	0.588	2	0.533
Error	31		31		31		99	

**Notes.**

** *P*-value < 0.01 (2-tailed).

*** *P*-value < 0.001 (2-tailed).

**Table 4:** Summary of ANCOVAs for the clutch size/egg size investment trade-off and reproduction/growth investment trade-off of female *P. nigromaculatus* in mesocosm experiments. With temperature as a fixed factor, using feeding rate and temperature × feeding rate as covariates.

Source of variation	Clutch/egg investment trade-off		Reproduction/growth investment trade-off	
	*df*	*F*	*df*	*F*
Feeding rate	1	20.339^***^	1	1.129
Temperature	2	1.305	2	2.296
Feeding rate × temperature	2	0.231	2	1.229
Error	31		31	

**Notes.**

*** *P*-value < 0.001 (2-tailed).

## Discussion

Reproductive parameters and body size changed due to the variation of temperature and feeding rate induced by controlled experiments in outdoor mesocosm treatments. When initial body size was controlled for, temperature was the major factor determining the reproductive time of *P. nigromaculatus*, which showed a significantly earlier onset under the temperature scenario of post-hibernation warming in both feeding rate treatments. Feeding rate was the major predictor of the reproductive status of individuals, clutch size, and the variation of body size for this species, which showed significantly positive correlation with feeding rate in any temperature treatments. Thus, our study suggests that reproduction and body size of amphibians might be affected by climate warming or food availability variation that caused by climate warming. In addition, we provide new experimental evidence on the allocation strategies of amphibian individuals for adjusting their reproductive output to cope with climate warming.

Because the reproductive output positively correlated with the body size of female frogs, inadequate sample sizes may not reflect the variation in clutch size. In this study, our samples covered a natural age structure of the species in each treatment, which were expected to be sufficient to reflect the reproductive variation. In addition, we used an appropriate feeding rate within expectations for this experiment. On one hand, we provided sufficient food for the frogs in these normal food treatments, as sometimes the larger ones did not consume all crickets at feeding time (Lardner & Loman, 2003); on the other hand, even in the low food treatments, the SVL of these frogs increased and some of them even bred successfully.

Recently, reports from the field have indicated that climate change has been pervasively affecting biological processes of many animals (Blaustein et al., 2010; Visser, 2013). Our results show compelling evidence that temperature rather than food availability is the main influencing factor for the reproductive time of female *P. nigromaculatus*. A previous study about male calling behavior (i.e., breeding behavior for males) on Korean Peninsula also indicated that temperature was a significant factor for male *P. nigromaculatus* (Yoo & Jang, 2012). This is possibly due to the fact that *P. nigromaculatus* is summer breeder independently of weather and with a typical “explosive” phenology (Wells, 1977; Fei, 1999). Amphibians, as ectothermic animals, have limited thermoregulation ability and they are exceedingly sensitive to climate change (Feder & Burggren, 1992; Blaustein et al., 2001; Lawler et al., 2010). Hence, the aggregated reproduction of this species may be induced by an environmental cue, such as temperature (Visser et al., 2010). This finding is consistent with those reports in lizards and birds, which showed that temperature could either affect reproductive events of side-blotched lizards (*Uta stansburiana*) (Tinkle & Irwin, 1965), or change timing of reproduction of great tits (*Parus major*) (Visser, Holleman & Caro, 2009). As an explosive breeder, the few limited breeding days of *P. nigromaculatus* are considered as the key determinant of life-history traits (Kozlowski & Teriokhin, 1999). Therefore, these changes on breeding phenology induced by temperature would influence reproductive success and allocation trade-off pattern of this species. Furthermore, amphibian metabolism is positively correlated with temperature (Gillooly et al., 2001; Dillon, Wang & Huey, 2010). When the food resource is fixed, there should be a higher energy consumption for metabolism under a warmer condition (Dillon, Wang & Huey, 2010), and as a result, there is a lower energy investment in reproduction or growth. However, no effect of temperature on reproduction or growth parameters except reproductive time was found during our experiments. Our result contrasts with the experimental reports in tadpoles, which showed complex results where they either reduced or increased in size at metamorphosis under warmer temperatures (Álvarez & Nicieza, 2002). Although in a suitable temperature regime, tadpoles in a better food resource could reach a larger size (Álvarez & Nicieza, 2002), we did not find this phenomenon in adult frogs. These differences could be caused by the different developmental constraints between tadpoles and adult frogs (Warne & Crespi, 2015).

The variation of food resource, as an indirect effect of climate change, could affect the reproduction of animals through food webs. In our experiment, some small individuals not even breed successfully, since they invested proportionately less on reproduction than larger ones, and spent more in growth to obtain a better fitness following the Cope’s rule (Rensch, 1948). Moreover, the clutch size of *P. nigromaculatus* also showed a positive correlation with body size, regardless of temperature scenarios treatments. Such correlations between body size and clutch size and/or egg size usually appeared among amphibians (Lardner & Loman, 2003; Wang et al., 2009; Xu & Li, 2013), reptiles (Brown & Shine, 2002; Sun et al., 2013) and other taxa (Grossman, McDaniel & Ratajczak Jr, 2002). After correcting for initial body size, the experiment showed that individuals in normal food treatment displayed better reproductive success, bigger clutch size, and larger body size than those in low food treatments in either temperature scenario. In contrast, egg size was not affected by feeding rate. Therefore, under a limited food resource, females tended to reduce investment or skip reproduction altogether in the upcoming season, and instead, invested residual energy to the following season for a higher fecundity, as found in previous studies of amphibians and reptiles (Loman, 1978; Schwarzkopf, 1993). On the other hand, some frogs could reserve a portion of or all eggs after reproduction (Loman, 1978; Reyer, Frei & Som, 1999), and use these unlaid eggs as stored energy reserves for reproduction in the following breeding season (Lardner & Loman, 2003). Considering that the experimental frogs of control treatments mated and bred successfully in synchrony with the wild population, hence, the decrease in clutch size or skipping in reproduction of experimental individuals may be due to the similar allocation strategy in this species. It means that we should account for the actual investment in reproduction during several successive breeding seasons (Lardner & Loman, 2003).

The negative correlation between clutch size and egg size was documented in previous studies (Warne & Charnov, 2008; Wang et al., 2009) and this was concluded as a universal ecological rule in life-history traits regardless of reproductive mode among many amphibians (Duellman & Trueb, 1986; Wells, 2010). There was also a trade-off in allocation between clutch size and egg size investment on female pond frogs. Our results showed that under good food resource conditions the frogs tended to allocate more investment in clutch size rather than egg size. In addition, our experiment suggested that there was a lack of trade-off in allocation between reproduction and growth investment on female pond frogs. Investments in both reproduction and growth were higher in normal food treatments than low food treatments regardless of any temperature scenarios. Another study showed that there was also no trade-off between reproduction and growth in adult female *R. temporaria* (Lardner & Loman, 2003). The lack of trade-off might be explained by the Y-model theory (Van Noordwijk & De Jong, 1986). The theory argued that a huge variation in acquisition (e.g., food availability) could mask a weak allocation (e.g., reproduction/growth investment) among individuals (King, Roff & Fairbairn, 2011). In our experiment, the food availability between high and low food treatments is up to approximately 3:1. This large advantage in food resource could allow the experimental frogs in high food treatments to allocate more resources in both reproduction and growth, rather than to make a trade-off in investment allocation between them. The flexible allocation strategy offers another explanation for the lack of trade-off (Warne et al., 2012). As it is expected that adult individuals find hibernation sites more efficiently than young ones, age and individual experience might influence the timing of hibernation, although such hypothesis should be further tested.

Although the species have to spend more energy in metabolism and maintenance under warmer condition (Gillooly et al., 2001), amphibians also have some thermoregulation ability to avoid energy consuming through behavioral adjustment (Pough, 1980). On the other hand, climate warming can also lead an extended season for growing and the frogs could obtain more energy for growth and reproduction due to the continuous food supply. Hence, both of the aspects could alter the life-history traits and further complicate the trade-off in energy allocation under climate warming. In addition, an appropriate choice of parameter may be crucial (Doughty & Shine, 1997), which could non-destructively measure and assess the actual energy invested in growth for the following reproduction when taking into account behavioral thermoregulation and variable food supply.

In conclusion, our study has demonstrated experimental evidence for food availability, rather than temperature, as a direct factor affecting the reproductive parameters and the variation in body size for *P. nigromaculatus*. Although experimental warming can change the reproductive phenology of the species, it did not show an effect on the trade-off allocation of growth vs. reproduction and clutch size vs. egg size. Therefore, our results suggest that the uncertain variation in food availability caused by current climate warming could lead to more complex effects on the life-history of amphibians. These less predictable responses of amphibians may require more thorough species protection plans for dealing with ongoing climate change.

## Supplemental Information

10.7717/peerj.1326/supp-1 Supplemental Information 1 Supporting Information Experimental operating details and detailed results.

10.7717/peerj.1326/supp-2 Supplemental Information 2 Raw data of the article (details in supplementary files)

## Additional Information and Declarations

### Field Study Permissions

The following information was supplied relating to field study approvals (i.e., approving body and any reference numbers): The experiments were carried out in accordance with the guidelines for the Use of Animals in Research issued by Institute of Zoology, Chinese Academy of Sciences (Project No. 2011/43).
