# Supplementary material for: Allocation trade-off under climate warming in experimental amphibian populations
Source: PeerJ. 2015 Oct 20;3:e1326. doi: 10.7717/peerj.1326 (PMC4614843; doi:10.7717/peerj.1326)
Supplement: Supplemental Information 1 — Experimental operating details and detailed results. [file peerj-03-1326-s001.pdf]

Supporting Information

Allocation trade-off under climate warming in experimental amphibian populations

Xu Gao, Changnan Jin, Arley Camargo and Yiming Li[1*]

Experimental operating details:

Experimental warming details

During the experimental warming periods in PreW, PreW-LF, PostW and PostW-LF treatments, the tops of each mescosm unit were covered with ethylene vinyl acetate films (EVA, thickness: 0.1 mm) with holes (diameter: 25 mm, 8 holes m^{-2}) for consistency in precipitation. After warming and during the remaining experimental period, the tops of these treatments were changed to insect screens (40 mesh) for protection. In the other treatments (CG and LF), the tops were also covered with insect screens during the entire experimental periods. There was no difference in rainfall between the two kinds of top covers (Gao et al., 2015). Air humidity was on average 81.3 ± 8.1% (mean ± SD) in the EVA tops treatments and 79.6 ± 9.1% in the insect screens tops treatments.

Air temperature controlled details

The daily fluctuating air temperature was controlled by an equipment, which consisted of a louvered exhaust fan for expelling air and a temperature difference controller for measuring temperature and controlling the fan. When the air temperature in pre- or post-hibernation warming treatment was 4 °C higher than ambient temperature treatment, a temperature difference controller activated a louvered exhaust fan to expel the warmed air and to reduce the air temperature until 1 °C higher; then the temperature difference controller closed the fan and the greenhouse warmed the air until reached 4 °C higher again, repeating the previous cycle.

References:

Gao, X., C. Jin, D. Llusia & Y. Li (2015) Temperature-induced shifts in hibernation behavior in experimental amphibian populations. *Sci Rep-Uk,* **5,** 11580.

Table S1. Summary of ANCOVAs for the reproduction parameters and growth of *P. nigromaculatus* in mesocosm experiments, with temperature as fixed effects, replicates as random factor, and using feeding rate, temperature × feeding rate and initial body size as covariates.

Source of variation	Reproductive timing		Clutch size (log_{10}-transformed)		Egg size		Variation of body size	
	df	*F*	*df*	*F*	*df*	*F*	*df*	*F*
Temperature	2	12.494***	2	0.291	2	0.866	2	0.597
Feed rate	1	0.014	1	43.777***	1	1.393	1	67.547***
Temperature × Feeding rate	2	0.592	2	0.543	2	0.768	2	0.519
Initial body size	1	6.990*	1	543.203***	1	174.689***	1	0.569
Replicates	2	1.602	2	2.502	2	2.336	2	0.520
Error	28		28		28		98	

* *P*-value < 0.05 (2-tailed); ** *P*-value < 0.01 (2-tailed); *** *P*-value < 0.001 (2-tailed).

Table S2. Summary of ANCOVAs for the variation of weight and SVL for *P. nigromaculatus* in mesocosm experiments, with temperature as fixed effects, and using feeding rate, temperature × feeding rate and initial body size as covariates.

Source of variation	With replicates as random factor				Without replicates as random factor			
	Finial weight		Finial SVL		Finial weight		Finial SVL	
	df	*F*	*df*	*F*	*df*	*F*	*df*	*F*
Temperature	2	0.659	2	0.214	2	0.626	2	0.21
Feed rate	1	88.807***	1	12.092***	1	88.758***	1	12.423***
Temperature × Feeding rate	2	0.275	2	0.805	2	0.3	2	0.847
Initial weight/SVL	1	511.803***	1	664.822***	1	516.571***	1	680.673***
Replicates	2	0.818	2	0.081	-	-	-	-
Error	96		96		98		98	

*** *P*-value < 0.001 (2-tailed).

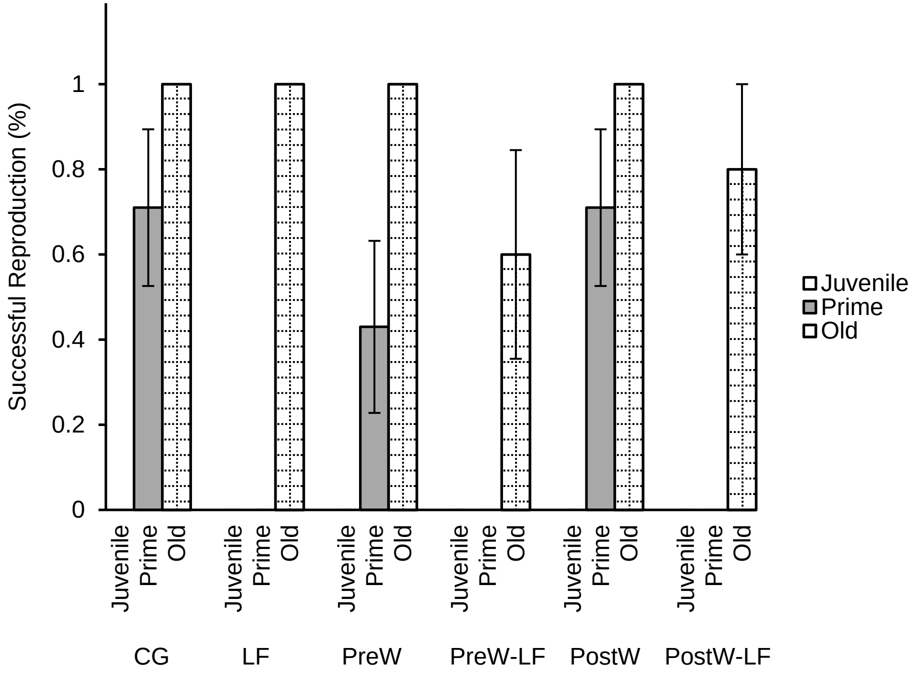

Figure S1 The reproductive status of different age classes of pond frogs among experimental treatments: control group (CG), low food treatment (LF), pre-hibernation warming with normal (PreW) or low food level treatment (PreW-LF), post-hibernation warming with normal (PostW) or low food level treatment (PostW-LF). Open bars indicated juvenile-age (< 2 years), dark bars indicated prime-age (2 – 3 years) and grid pattern bars indicated old-age (≥ 4 years)

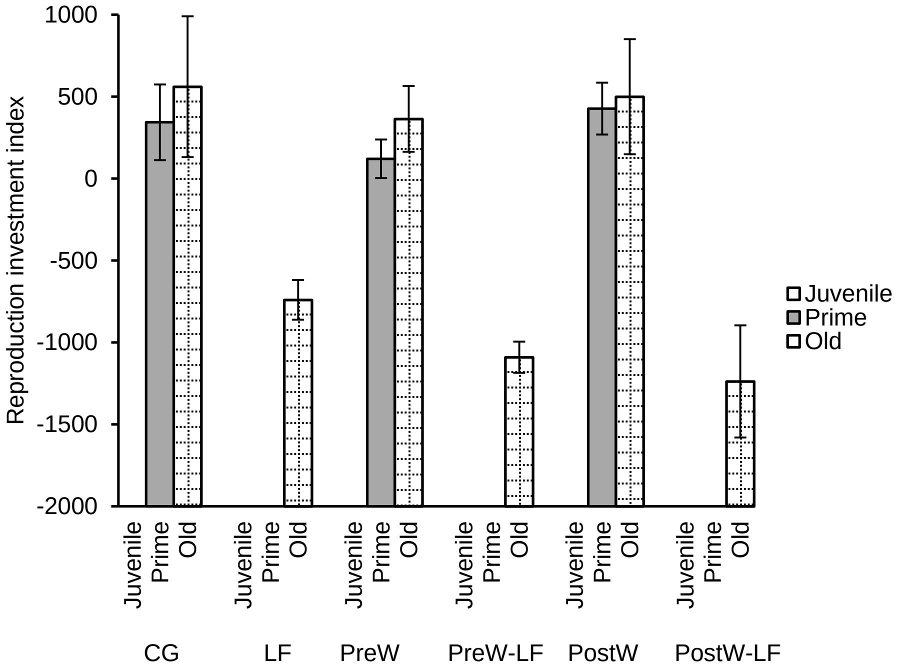

Figure S2 The reproduction investment index of different age classes of pond frogs among experimental treatments: control group (CG), low food treatment (LF), pre-hibernation warming with normal (PreW) or low food level treatment (PreW-LF), post-hibernation warming with normal (PostW) or low food level treatment (PostW-LF). Open bars indicated juvenile-age (< 2 years), dark bars indicated prime-age (2 – 3 years) and grid pattern bars indicated old-age (≥ 4 years)

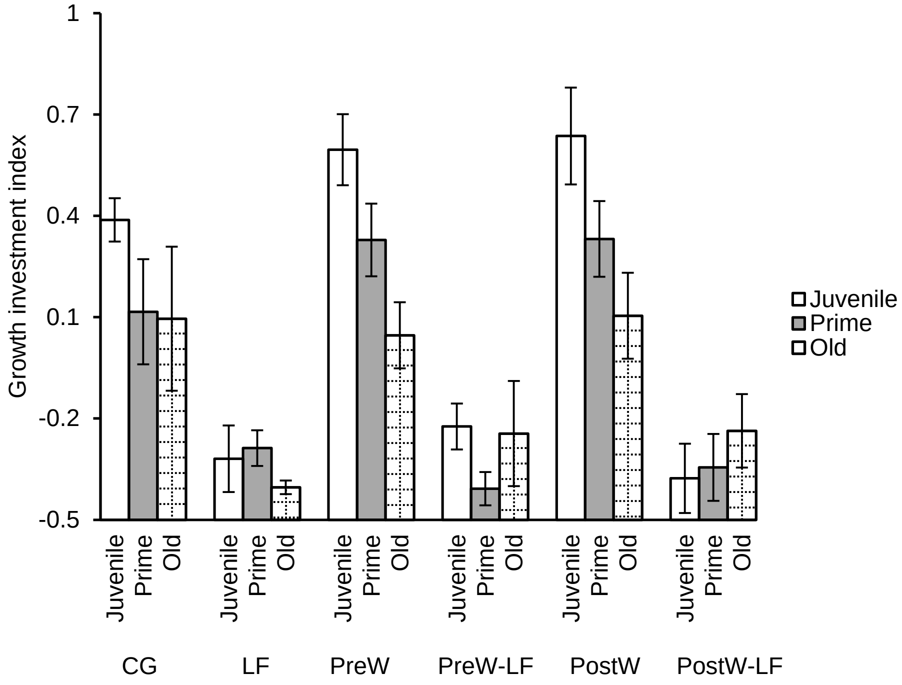

Figure S3 The growth investment index of different age classes of pond frogs among experimental treatments: control group (CG), low food treatment (LF), pre-hibernation warming with normal (PreW) or low food level treatment (PreW-LF), post-hibernation warming with normal (PostW) or low food level treatment (PostW-LF). Open bars indicated juvenile-age (< 2 years), dark bars indicated prime-age (2 – 3 years) and grid pattern bars indicated old-age (≥ 4 years)
