# Supplementary material for: Allocation trade-off under climate warming in experimental amphibian populations
Source: PeerJ. 2015 Oct 20;3:e1326. doi: 10.7717/peerj.1326 (PMC4614843; doi:10.7717/peerj.1326)
Supplement: Supplemental Information 2 [file peerj-03-1326-s002.pdf]

Raw data of "Allocation trade-off under climate warming of an experimental amphibian population"

Xu Gao, Changnan Jin, Arley Camargo and Yiming Li*

Treatments	Breeding	Reproductive timing (day)	Clutch size	Egg size (mm)	Age (Year)	Initial SVL (mm)	Initial weight (g)	Final SVL (mm)	Final weight (g)	Consuming cricket number
Control group	No	-	-	-	1	52.27	14.3	57.23	18.4	157
Control group	No	-	-	-	1	53.38	16.6	60.24	22	166
Control group	No	-	-	-	1	51.94	16.4	59.88	17.3	138
Control group	No	-	-	-	1	49.54	12.4	57.55	16.8	137
Control group	No	-	-	-	2	56.9	18.3	63.57	23.9	153
Control group	No	-	-	-	1	56.04	17.5	61.22	18	140
Control group	No	-	-	-	2	58.26	24.6	64.19	28.5	288
Control group	No	-	-	-	1	45.09	9.6	56.61	15.9	138
Control group	No	-	-	-	1	54.5	17.7	60.27	19.1	157
Control group	Yes	122	939	1.60	2	60.47	24.1	63.13	14.8	308
Control group	Yes	119	2583	1.54	5	75.48	53.4	76.98	33.9	405
Control group	Yes	106	2865	1.53	5	77.97	47.3	81.73	38.1	414
Control group	Yes	127	1092	1.60	3	65.85	32.2	68.56	23.4	258
Control group	Yes	119	793	1.63	3	62.97	28.7	62.99	22.4	270
Control group	Yes	115	4553	1.51	6	81.34	67.9	83.85	41.6	536
Control group	Yes	116	881	1.60	3	63.1	30.2	68.27	24.6	266
Control group	Yes	125	723	1.62	2	58.31	20.3	62.62	15.9	133
Low food group	No	-	-	-	1	51.6	13.6	56.28	12.2	46
Low food group	No	-	-	-	2	57.47	20	58.72	13	46
Low food group	No	-	-	-	3	62.87	27.4	63.63	18.2	91
Low food group	No	-	-	-	2	58.42	19.6	59.95	15.4	47
Low food group	No	-	-	-	1	53.78	19.9	59.48	13.1	48
Low food group	No	-	-	-	2	56.67	19.1	59.8	13.9	48
Low food group	No	-	-	-	3	65.83	27.5	70.44	16.4	89
Low food group	No	-	-	-	2	58.5	25.5	62.28	16.9	90
Low food group	No	-	-	-	2	57.75	19.5	59	13.7	52
Low food group	No	-	-	-	2	56	25.4	61.92	16.7	95
Low food group	No	-	-	-	3	61.87	30.9	63.04	17.3	88
Low food group	No	-	-	-	1	51.94	18.5	53.53	11.7	48
Low food group	No	-	-	-	1	47.5	12.6	49.71	9.9	47
Low food group	No	-	-	-	2	54.86	18.6	62.91	12.9	50
Low food group	Yes	117	1528	1.53	4	72.38	41.2	73.46	24.4	143
Low food group	Yes	126	1206	1.56	5	67.27	36.8	67.47	22.6	91
Low food group	Yes	112	851	1.59	5	66.46	33.9	67.29	20.9	93
Post-hibernation warming group	No	-	-	-	1	52.94	15.1	62.53	21.2	158
Post-hibernation warming group	No	-	-	-	2	58.53	21.9	62.46	27.5	328
Post-hibernation warming group	No	-	-	-	2	58.14	24	63.88	31.2	296
Post-hibernation warming group	No	-	-	-	1	50.92	12.5	64.17	20.1	170
Post-hibernation warming group	No	-	-	-	1	49.35	12.6	53.42	13.5	144
Post-hibernation warming group	No	-	-	-	1	48.47	11.9	60.65	18.1	156
Post-hibernation warming group	No	-	-	-	1	52.66	12.2	60.74	20.1	152
Post-hibernation warming group	No	-	-	-	1	51.54	13.6	65.47	19.3	155
Post-hibernation warming group	No	-	-	-	1	50.73	12.5	57.12	16.8	141
Post-hibernation warming group	Yes	113	971	1.60	3	65.23	28.9	69.82	24.6	284
Post-hibernation warming group	Yes	98	2905	1.49	5	77.58	56.6	78.47	44.1	441
Post-hibernation warming group	Yes	105	2390	1.55	4	72.36	42.4	72.72	34.8	453
Post-hibernation warming group	Yes	111	891	1.60	3	62.05	23.7	66.81	20.8	318
Post-hibernation warming group	Yes	105	4104	1.50	6	79.72	69.9	79.92	42.8	564
Post-hibernation warming group	Yes	90	850	1.60	5	57.36	21.8	64.48	17.1	262
Post-hibernation warming group	Yes	116	894	1.61	3	62.98	29.5	63.25	24.8	276
Post-hibernation warming group	Yes	102	826	1.64	3	61.67	24.1	65.74	22	302
Post-hibernation warming group	Yes	122	607	1.64	3	57.33	20.4	67.08	17.2	286
Post-hibernation warming with low food group	No	-	-	-	3	63.55	27	65.59	19.9	104
Post-hibernation warming with low food group	No	-	-	-	3	64.69	20.5	70.43	14.9	95
Post-hibernation warming with low food group	No	-	-	-	1	53.71	16.2	57.43	10.5	53
Post-hibernation warming with low food group	No	-	-	-	1	45.96	13.6	55.08	10.2	49
Post-hibernation warming with low food group	No	-	-	-	2	56.44	15.6	56.93	10.8	50
Post-hibernation warming with low food group	No	-	-	-	1	54.79	19.9	57.91	11.3	43
Post-hibernation warming with low food group	No	-	-	-	2	60.76	23.8	61.53	14.1	114
Post-hibernation warming with low food group	No	-	-	-	3	63.99	26.8	64.13	17.9	94
Post-hibernation warming with low food group	No	-	-	-	2	57.65	18.9	57.95	10	51
Post-hibernation warming with low food group	No	-	-	-	4	66.38	32	66.58	18.6	107
Post-hibernation warming with low food group	No	-	-	-	1	52.11	14.5	52.6	9.2	48
Post-hibernation warming with low food group	No	-	-	-	2	59.46	19.5	59.93	11.8	51
Post-hibernation warming with low food group	No	-	-	-	2	57.69	23.2	63.29	18.3	99
Post-hibernation warming with low food group	No	-	-	-	1	55.1	14.3	55.21	11.5	50
Post-hibernation warming with low food group	Yes	99	1590	1.57	4	73.68	41.3	76.04	22.7	150
Post-hibernation warming with low food group	Yes	102	1255	1.54	6	70.21	42.3	70.51	27.4	134
Post-hibernation warming with low food group	Yes	92	1563	1.54	5	76.24	54	79.27	34.9	135
Post-hibernation warming with low food group	Yes	115	782	1.60	4	65.73	32.7	70.33	23.2	93
Pre-hibernation warming group	No	-	-	-	1	53.95	20.6	62.24	25	340
Pre-hibernation warming group	No	-	-	-	3	64.8	26.1	67.2	30.2	292
Pre-hibernation warming group	No	-	-	-	1	47.29	12.9	56.56	15.5	141
Pre-hibernation warming group	No	-	-	-	1	52.87	15.4	64.73	20.2	148
Pre-hibernation warming group	No	-	-	-	1	54.27	15.7	60	21	157
Pre-hibernation warming group	No	-	-	-	2	57.91	20.9	60.92	25.3	310
Pre-hibernation warming group	No	-	-	-	2	54.54	15.3	63.77	21.8	165
Pre-hibernation warming group	No	-	-	-	2	57.63	17.4	62.11	21.7	152
Pre-hibernation warming group	Yes	108	2580	1.55	4	73.96	40.6	74.52	35.2	459
Pre-hibernation warming group	Yes	121	2881	1.52	4	76.51	51.5	79.78	32.4	435
Pre-hibernation warming group	Yes	125	1451	1.57	2	69.07	36.1	70.24	28.5	294
Pre-hibernation warming group	Yes	107	3052	1.52	5	78.22	52.2	78.86	32	438
Pre-hibernation warming group	Yes	107	2656	1.54	4	74.18	46.5	76.99	34.5	441
Pre-hibernation warming group	Yes	122	932	1.60	3	64.8	26.1	67.2	21	314
Pre-hibernation warming group	Yes	118	1235	1.58	3	65.12	31.7	65.17	29.6	304
Pre-hibernation warming group	Yes	115	811	1.62	4	61.92	27.7	67.36	23.1	270
Pre-hibernation warming group	Yes	118	1473	1.56	5	64.6	33.7	71.92	24.4	278
Pre-hibernation warming group	Yes	105	4262	1.47	6	85.26	73.7	86.52	41.7	556
Pre-hibernation warming with low food group	No	-	-	-	1	50.8	15.5	56.01	12.6	53
Pre-hibernation warming with low food group	No	-	-	-	6	76.79	62.4	78.57	43.6	189
Pre-hibernation warming with low food group	No	-	-	-	3	64.58	25.8	65.35	16.8	102
Pre-hibernation warming with low food group	No	-	-	-	1	50.96	13.9	57.54	12.2	54
Pre-hibernation warming with low food group	No	-	-	-	1	48.77	12.8	49.87	9.8	49
Pre-hibernation warming with low food group	No	-	-	-	1	54.27	18.9	58.46	12.9	51
Pre-hibernation warming with low food group	No	-	-	-	5	71.12	41	74.27	28.2	142
Pre-hibernation warming with low food group	No	-	-	-	2	60.56	25	62.03	15.8	97
Pre-hibernation warming with low food group	No	-	-	-	3	63.3	30.1	64.08	16.6	96
Pre-hibernation warming with low food group	No	-	-	-	1	54.8	20.6	58.91	14.6	98
Pre-hibernation warming with low food group	No	-	-	-	1	50.15	14.2	57.05	11.3	50
Pre-hibernation warming with low food group	No	-	-	-	2	55.4	22.3	57.78	14.6	108
Pre-hibernation warming with low food group	No	-	-	-	1	54.48	18.7	56.89	12.9	50
Pre-hibernation warming with low food group	No	-	-	-	2	60.69	25.7	60.95	20	98
Pre-hibernation warming with low food group	Yes	111	1606	1.53	5	71.94	43.4	72.05	21.4	140
Pre-hibernation warming with low food group	Yes	117	783	1.60	4	68.64	33.8	68.84	20.6	95
Pre-hibernation warming with low food group	Yes	121	1108	1.53	4	66.77	43	72.71	24	146
